# Testicular versus ejaculated sperm should be used for intracytoplasmic sperm injection (ICSI) in cases of infertility associated with sperm DNA fragmentation | *Opinion: Yes*

**DOI:** 10.1590/S1677-5538.IBJU.2018.04.03

**Published:** 2018

**Authors:** Sandro C. Esteves

**Affiliations:** 1 ANDROFERT, Andrology and Human Reproduction Clinic, Campinas, SP, Brasil;; 2 Departamento de Cirurgia (Disciplina de Urologia), Faculdade de Ciências Médicas, Universidade de Campinas (UNICAMP), Campinas, SP, Brasil;; 3 Faculty of Health, Aarhus University, 8000 Aarhus C, Denmark

**Keywords:** Semen, Infertility, Male, Sperm Injections, Intracytoplasmic, Sperm DNA Fragmenttion, Testicular Sperm

The use of testicular in preference over ejaculated sperm for intracytoplasmic sperm injection (ICSI) has gained increased attention due to reports of better pregnancy outcomes using testicular sperm for cases of infertility associated with high sperm DNA fragmentation (SDF) (reviewed by Esteves et al. (1)). Indeed, it has been a common practice to perform testicular sperm retrieval for ICSI (Testi-ICSI) in selected groups of non-azoospermic men. In a recent survey study involving infertility experts from 19 countries, 67% responders admitted that an abnormal SDF test result would affect their decision to utilize testicular instead of ejaculated sperm for ICSI (2). Interestingly, identical numbers were reported by attendees of an interactive debate session held during the 2017 annual meeting of the American Society for Reproductive Medicine (unpublished data).

The matter concerned has been subjected to opinionated debate as Testi-ICSI represents a paradigm shift in clinical practice (1, 3-5). I defend the argument that *“Infertile couples undergoing ICSI, whose male partners have elevated SDF levels in the neat ejaculate, should be offered testicular sperm in the next ICSI cycle, provided SDF is persistent after treatment of the underlying condition, or the clinical scenario does not allow treatment”.* There are three essential, evidence-based premises supporting this clinical approach, which I will discuss in the next paragraphs.

First, SDF not only impacts *in vitro* fertilization (IVF) and ICSI pregnancy outcomes but also contributes to pregnancy loss. In fact, results from the most recent and largest systematic review and meta-analysis about the impact of SDF on assisted reproductive technology (ART), which pooled data from 70 studies and over 17,000 IVF and ICSI cycles, indicate that SDF reduces the probability of a successful pregnancy following ART (6). This observation holds true for both IVF and ICSI studies (IVF studies: odds ratio [OR] 1.15, 95% confidence interval [CI] 1.05-1.27; P=0.003; ICSI studies: inverse OR 1.12, 95% CI 1.01-1.25, P=0.02) and the four most common assays (TUNEL-terminal deoxynucleotidyl transferase dUTP nick end labeling, SCD-sperm chromatin dispersion, Comet-single cell gel electrophoresis, and SCSA-sperm chromatin structure assay) utilized for SDF assessment. Notably, the magnitude of effect size was amplified when female infertility factors were excluded (1704 cycles, OR 1.37, 95% CI 1.11-1.68, P=0.003), thus stressing the importance of the male factor concerning SDF.

Along the same lines, the risk of miscarriage is increased in couples with high SDF subjected to IVF and ICSI. Of the systematic reviews with meta-analysis, Robinson et al. aggregated the evidence of 16 studies and showed a significant increase in miscarriage rates in couples whose male partners had high SDF compared with those with low SDF (Relative Risk [RR] 2.2, 95% CI 1.54-3.03, P<0.00001) (7). Likewise, Zhao et al. pooled data from over 2,500 couples and showed that SDF had a detrimental effect on pregnancy after IVF/ ICSI (All studies: OR 2.3; 95% CI 1.55-3.35, P<0.01; ICSI studies only: OR 2.7, 95% CI 1.40-5.14, P=0.003) (8). Despite using different SDF assays and not controlling for all confounding factors, both studies concluded that SDF testing should be offered to couples following IVF/ICSI failure, which is consonant with the recommendations of the recent clinical practice guidelines (CPG) on SDF testing issued by the Society for Translational Medicine (9). In practical terms, the OR of 2.7 means that an IVF Clinic performing 1,000 ICSI cycles a year with an overall clinical pregnancy rate (CPR) of about 40% will lose 82 pregnancies as a result of SDF, which means an absolute pregnancy reduction of 21%.

Second, testicular sperm have lower DNA fragmentation than ejaculated counterparts in men with elevated SDF in the neat ejaculate. This conclusion derives from a recent systematic review and meta-analysis, including five studies and 143 patients who served as their control, that is, SDF was measured in ejaculated and testicular specimens obtained from the same men (10). Four studies used the TUNEL assay whereas one study used the SCD assay. Overall, SDF rates were markedly lower in testicular than ejaculated sperm (Mean Difference [MD] −24.6%, 95% CI −32.5% to −16.6%, P<0.00001). Notably, the consistency in the direction of estimates -favoring testicular sperm in all studies- adds confidence to these findings (Figure-1).

**Figure 1:**
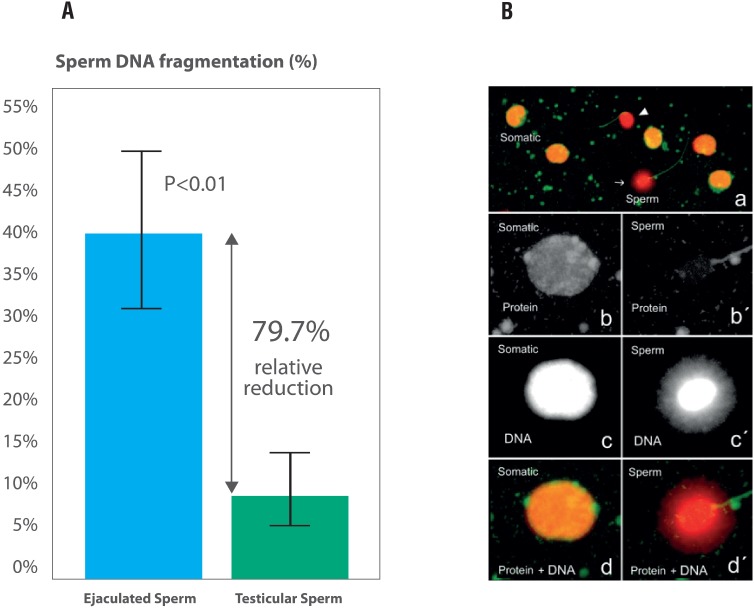
Comparison of sperm DNA fragmentation rates in ejaculated and testicular sperm of 81 infertile men undergoing icsI: (A) Use of testicular sperm for icsi resulted in an absolute reduction of 32.6% (relative reduction of 79.7%) in SDF; (B) Sperm chromatin dispersion (SCD) test for assessing SDF in testicular sperm. A variant of the Halosperm test (Halotech DNA, Spain) that combines a dual fluorescent cocktail probe to discriminate somatic cells from spermatozoa was used. Spermatozoa and somatic cells exhibit differences in the wavelength emission associated with each fluorochrome (green for proteins and red for DNA). Spermatozoa exhibit only red fluorescence on the sperm head owing to protamine removal, while non-sperm cells fluoresce yellow as a result of the combined emission of both fluorochromes (A). Spermatozoa exhibiting red fluorescence with a green flagellum and no halo of chromatin dispersion represented those with fragmented DNA (arrow cap). In contrast, spermatozoa exhibiting red fluorescence with a green flagellum and haloes of chromatin dispersion represented those with non-fragmented DNA (arrow). A somatic cell with its typical high protein and DNA contents and a spermatozoon with its characteristic low protein remnant and high DNA content are seen in B and c, respectively, using a single channel fluorescence emission. After merging the information provided by protein and DNA selective staining, somatic cells and spermatozoa can be easily distinguished (d and d'). In addition, the sperm tail fluoresces in green, and this feature also helps to distinguish spermatozoa from other cell elements (a and d'). Adapted with permission from Esteves et al. (11).

A study from our group, included in the meta-analysis mentioned above, compared DNA fragmentation rates between ejaculated and testicular sperm in 81 men with idiopathic oligozoospermia and elevated DFI (11). In our study, SDF rates by SCD using fluorescence microscopy were about 80% lower in testicular than ejaculated sperm (Figure-2) (Ejaculate: 40.7% ± 9.9%; Testis: 8.3% ± 5.3%, P<0.001). This study as well as others (12-15), included in that meta-analysis, provided data to answer the question of how often testicular specimens are better than ejaculated specimens concerning SDF (Table-1). The answer is that SDF is lower in testicular than ejaculated sperm in virtually all men with high SDF levels in semen, a reassuring data for the use of testicular sperm.

**Figure 2:**
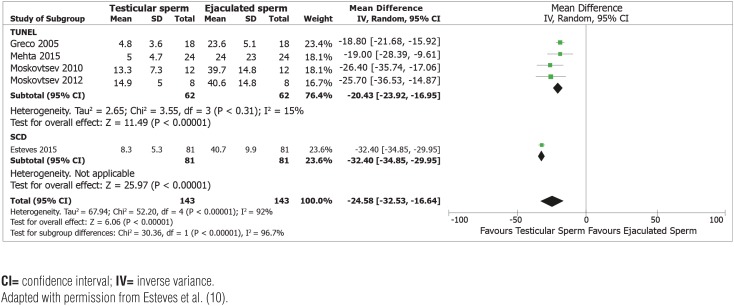
Forest plot showing mean difference for sperm DNA fragmentation (SDF) rates between testicular and ejaculated sperm in men with high SDF, including subgroup analysis according to SDF assay (terminal deoxynucleotidyl transferase dUTP nick end labeling (TUNEL) and sperm chromatin dispersion (SCD)).

**Table 1:** Characteristics of studies comparing sperm DNA fragmentation rates between testicular and ejaculated sperm of the same men and how often SDF rates were lower in testis versus ejaculated sperm among men with high SDF in semen.

Study	Infertile male population studied	No. patients	SDF assay	DFI cutoff (%)	No. patients (%) SDF lower in testicular sperm than ejaculated sperm
Greco et al. 2005 (15)	Non-smokers; Mean sperm count: 26.8 M/mL; Sperm motility: 36.7%; Sperm morphology: 20.9%	18	TUNEL	15	17 (94.5%)
Moskovtsev et al. 2010 (13)	High DFI despite AOX	12	TUNEL	30	11 (91.7%)
Esteves et al. 2015 (11)	Idiopathic oligozoospermia (5-15 M/mL); high DFI despite AOX	81	SCD	30	81 (100.0%)

**DFI =** DNA fragmentation index; **SDF =** Sperm DNA Fragmentation; **AOX =** Oral antioxidant therapy; **TUNEL =** terminal deoxynucleotidyl transferase dUTP nick end labeling; **SCD =** sperm chromatin dispersion

One of the main reasons why SDF is higher in semen than testis relates to the susceptibility of sperm chromatin to oxidative attack, particularly during epididymis transit (16). Apoptosis triggered by testicular conditions and by oxidative stress during sperm transit through the male reproductive tract can explain the high positivity of ejaculated sperm from infertile men for SDF, a phenomenon observed in both animal and human studies (17, 18). The source of the oxidative stress can be anything from a specific clinical condition such as a varicocele and a subclinical genital infection to age, obesity, smoking, and environmental exposure to toxicants (19). This oxidative-induced damage to sperm chromatin can be avoided in selected ICSI candidates provided the epididymis is bypassed.

Lastly, the existing evidence indicates that sperm retrieved from the testis of men with elevated SDF result in higher pregnancy rates when used for sperm injections. In the meta-analysis discussed above, we also looked at ICSI outcomes using testicular versus ejaculated sperm in men with confirmed elevated SDF in semen (10). Four studies provided this data, including 507 cycles and 3,840 injected oocytes (11, 15, 20, 21). In three of the four studies, elevated SDF was defined by a DNA fragmentation index (DFI) of 29% or greater (Table-2). The odds ratio of achieving a clinical pregnancy using testicular sperm was 2.4 (95% CI 1.57-3.73, I^2^=34%, P<0.0001) and the results were conservative in subgroup analyses including couples with ICSI failure (OR 4.18, 95% CI 1.67-10.47, I^2^=36%, P=0.002) or first ICSI comers (OR 2.06, 95% CI 1.253.37, I^2^=39%, P=0.004). Furthermore, the OR of a live birth also favored testicular sperm (2.58, 95% CI 1.54-4.35, I^2^=0%, P=0.0003). Importantly, the odds of a miscarriage were reduced by approximately 67% overall using testicular sperm (OR 0.28, 95% CI 0.11-0.68, I^2^=11%, P=0.005) (Figure-3). The conclusion was that ICSI with testicular sperm improves reproductive outcomes when compared with ejaculated sperm in men with high SDF.

**Table 2:** Characteristics of studies comparing intracytoplasmic sperm injection (ICSI) outcomes using testicular versus ejaculated sperm in infertile men with high sperm DNA fragmentation (SDF) in semen.

Study	Design	Population	No. patients/cycles	SDF assay (cutoff)	ICSI Outcome
Greco et al. 2005 (15)	Case-control	ICSI failure (≥2); normozoospermia 1	18	TUNEL (15%)	2PN, CPR
Esteves et al. 2015 (11)	Prospective	Non-ICSI failure; oligozoospermia 2	172	SCD (30%)	2PN, CPR, miscarriage, LBR
Pabuccu 2016 (20)	Retrospective	ICSI failure (≥2); normozoospermia 2	71	TUNEL (30%)	2PN, CPR
Bradley et al. 2016 (21)	Retrospective	Non-ICSI failure; oligozoospermia 1	228	SCIT (29%)	2PN, CPR, miscarriage, LBR

1 The studied populations of Greco et al. and Bradley et al. were classified as normozoospermic (>15 million/mL) or oligozoospermic (<15 million/mL) based on the calculated mean or median sperm concentration.

2 The study by Esteves et al. and Pabuccu et al. included men with oligozoospermia (5-15 million/mL) and normozoospermia (>15 million/mL) based on the 2010 World Health Organization manual for semen analysis

**2PN** = two-pronuclear zygote; **TUNEL** = terminal deoxynucleotidyl transferase dUTP nick end labeling; **SCD** = sperm chromatin dispersion; **SCIT** = sperm chromatin integrity test, which is a variation of sperm chromatin sperm assay (SCSA); **CPR** = clinical pregnancy rate; **LBR** = live birth rate

**Figure 3:**
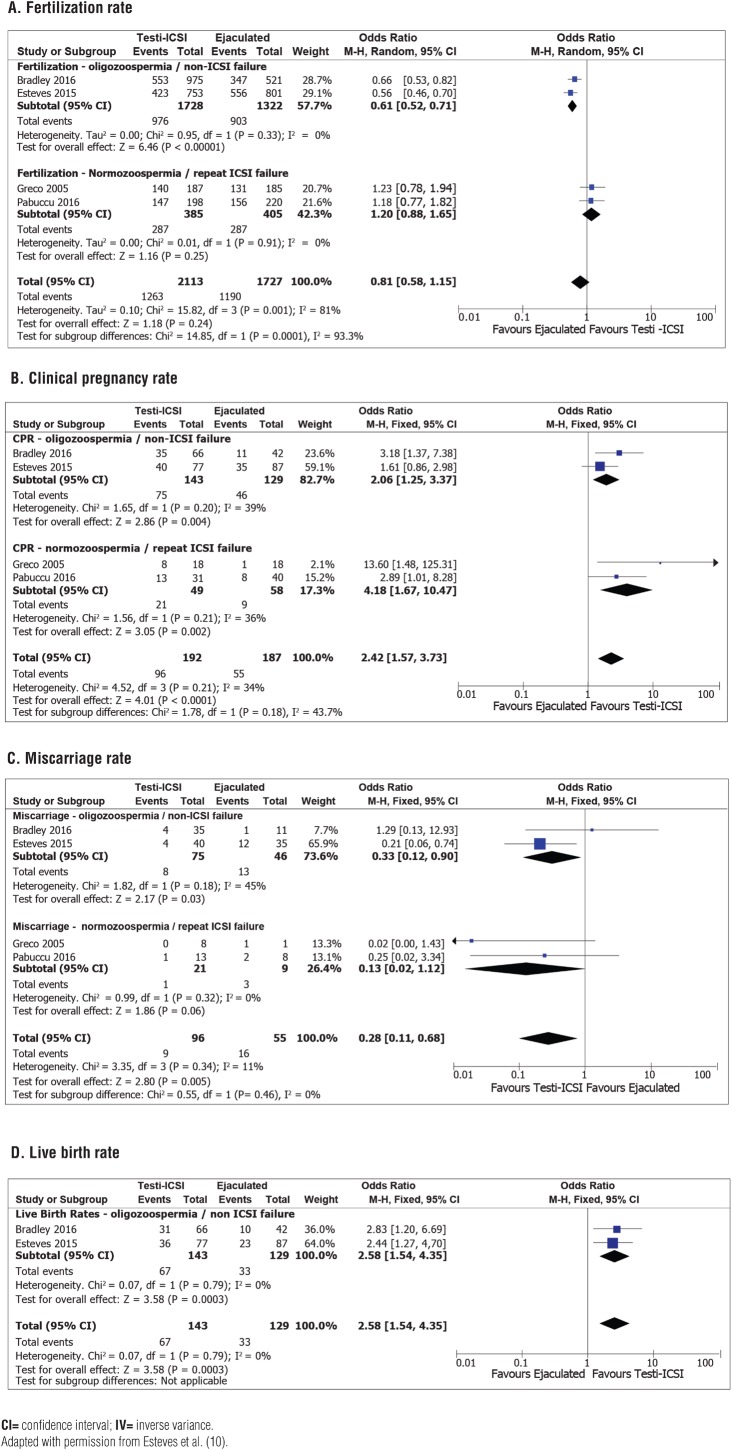
Forest plots showing odds ratios for (A) fertilization rates, (B) clinical pregnancy rates, (C) miscarriage rates, and (D) live birth rates with the use of intracytoplasmic sperm injection with testicular (Testi-ICSI) or ejaculated (Ejac-ICSI) sperm in men with high sperm DNA fragmentation, including subgroup analyses according to study population (repeated ICSI failure and non-ICSI failure) and semen analysis profile (oligozoospermia and normozoospermia).

A prospective, observational cohort study from our group (evidence level 2b), which had a substantial weight (59%) in the meta-analysis discussed above, included 147 infertile couples (11). The men had idiopathic oligozoospermia (5-15 million/mL) and persistently elevated SDF (DFI>30% by SCD) despite taking oral antioxidant therapy. The women were aged <40 years and had no apparent fertility issues. The main outcome measures were clinical pregnancy rate (CPR), live birth rate (LBR), and miscarriage rate, and the study was powered (80%) to detect a 30% difference in LBR between the groups with a significance level of 5%. The clinical characteristics of the couples subjected to ICSI using testicular versus ejaculated sperm were not statistically different. In this study, we found that LBR was significantly higher (P=0.007) in the Testi-ICSI group (46.7%) than in the Ejac-ICSI group (26.4%). Moreover, miscarriage rates were lower in couples who used testicular versus ejaculated sperm for ICSI (10% vs. 34.3%, P=0.012). The relative risk of achieving a live birth by Testi-ICSI was increased by 76% (RR 1.76, 95% CI 1.15-2.70). This means that the number needed to treat by Testi-ICSI compared with Ejac-ICSI to achieve one additional live birth was 4.9 (95% CI 2.8-16.8), thus suggesting that one out of five oocytes pick-ups can be avoided if testicular sperm is used in preference over ejaculated sperm.

Although the current data supporting testicular sperm for selected non-azoospermic infertile men is reassuring, it is important to recognize the existence of gaps in knowledge and the risks of sperm retrieval, as discussed in detail elsewhere (1). Briefly, there is still limited evidence as regards the clinical efficacy of Testi-ICSI. Furthermore, there is a need to define the best candidates for Testi-ICSI and compare its cost-effectiveness with other laboratory methods of sperm selection. Also, sperm retrieval has potential hazards, although the overall risk is low (<5%) and the complications minor (22, 23). These shortcomings, however, should not refrain from offering testicular sperm for selected ICSI couples provided we discuss with our patients the limitations of SDF testing and the possible clinical benefits and risks of Testi-ICSI.

In my practice, I request SDF testing to selected ART candidates and recommend treatment of the underlying conditions associated with SDF (24). Varicocele, lifestyle factors (smoking, obesity, occupational exposure), and genital infections are potentially correctable factors that have been associated with SDF. Identification and treatment of these conditions may decrease SDF and enable the use of ejaculated sperm for ICSI or the application of less complex assisted reproduction methods (reviewed by Esteves et al. (25)). Testi-ICSI is reserved for cases with persistently elevated SDF after all possible measures were taken to reduce SDF, or when the clinical scenario does not allow treatment. Importantly, we do not recommend Testi-ICSI to unselected populations of infertile men with untested SDF, such as those with cryptozoospermia, as the current evidence remains equivocal concerning the potential benefit of Testi-ICSI to this subset of men (26).

We rely on testicular sperm instead of laboratory methods to select specimens with lower SDF levels because it allows for sperm acquisition before transiting through the epididymis, which is when SDF is thought to be acquired. Also, it has been shown that Testi-ICSI provides higher LBR when compared with laboratory methods such as physiological intracytoplasmic sperm injection (PICSI) and intracytoplasmic morphologically selected sperm injection (IMSI) in couples with high SDF in semen (21). In a 2016 study, Bradley et al. evaluated 448 cycles in which sperm injections were carried out with ejaculated and testicular sperm. In the former, PICSI and IMSI were used to select sperm with better chromatin integrity for ICSI. They found that LBR birth rates with Testi-ICSI (49.8%) were significantly higher than IMSI (28.7%) and PIC-SI (38.3%). The lowest live birth rates (24.2%) were achieved when no method was used to select sperm for ICSI (P = 0.020).

Lastly, as for the health of offspring resulting from Testi-ICSI in cases of high SDF, there is lack of published data. However, reports of ICSI using testicular sperm in azoospermia have been overall reassuring, as no major differences are noted in the short-term neonatal outcomes and congenital malformation rates among children from fathers with nonobstructive azoospermia or obstructive azoospermia (27, 28).

In conclusion, I first presented evidence confirming that SDF negatively impacts ART pregnancy outcomes and is associated with pregnancy loss. Then, I provided data to substantiate the premise that SDF is lower in testicular than ejaculated sperm among infertile men with high SDF in semen. Lastly, I summarized the evidence supporting the proposition from which the use of testicular in preference over ejaculated sperm (therefore with lower SDF) is associated with improved ICSI pregnancy outcomes in couples whose male partners have high SDF in semen. These evidence-based premises make the argument stated at the beginning of this article “Infertile couples with ICSI failure should be offered Testi-ICSI if male partners have high SDF in the neat ejaculate” irrefutable. Therefore, Testi-ICSI should be considered in the treatment plan of infertile couples undergoing ICSI when the following conditions are met (i) Presence of high SDF levels in neat ejaculate, measured by a reliable assay with a validated threshold, and (ii) Persistence of elevated SDF levels despite treatment of the underlying condition causing SDF (if correctable). A failed ICSI cycle using ejaculated sperm with no other obvious reasons explaining that failure should reinforce consideration for the use of Testi-ICSI, provided the conditions mentioned above are met.

## DISCLOSURE

Expanded from an invited talk by the author delivered at the American Society for Reproductive Medicine (ASRM) Annual Meeting, San Antonio, USA, October 2017.
